# Synthesis of Hydrophilic and Amphiphilic Acryl Sucrose Monomers and Their Copolymerisation with Styrene, Methylmethacrylate and α- and β-Pinenes

**DOI:** 10.3390/ijms11041792

**Published:** 2010-04-16

**Authors:** Maria Teresa Barros, Krasimira T. Petrova, Raj P. Singh

**Affiliations:** 1 Departamento de Química,REQUIMTE, CQFB, Faculdade de Ciências e Tecnologia, Universidade Nova de Lisboa, 2829-516 Caparica, Portugal; 2 National Chemical Laboratory, Polymer Science & Engineering Division, Pune-411008, India; E-Mail: rp.singh@ncl.res.in

**Keywords:** vinyl sucrose ester monomers, biodegradable copolymers, free radical copolymerization, biodegradation culture test

## Abstract

Herein, we report the synthesis of monomethacryloyl sucrose esters, and their successful free radical homo- and co-polymerisation with styrene, methylmethacrylate, α-and β-pinene. The chemical, physical, structural and surface chemical properties of these polymers, containing a hydrophobic olefin backbone and hydrophilic sugar moieties as side chains, have been investigated. Biodegradation tests of the copolymer samples by a microbial fungal culture (*Aspergillus niger*) method showed good biodegradability. The chemical structure and surface chemistry of the synthesized homo- and co-polymers demonstrate their potential technological relevance as amphiphilic and biodegradable polymers.

## 1. Introduction

In 1946, Haworth and co-workers [1] reported the preparation of polymerisation products from substituted carbohydrates containing acrylate or methacrylate groups. Since then, there has been extensive interest in the synthesis and polymerisation of mono-functionalised carbohydrate monomers. Amphiphilic molecules, such as surfactants, lipids, copolymers, and proteins, find widespread use in the chemical, pharmaceutical, and food industry, because of their unique ability to self-assemble and modify surface/interfacial properties [2]. If natural resources such as polysaccharides are used as material polymers, their use as nanomaterials could expand further [3]. Some applications for sugar based polymers are drug delivery systems [4], dental medicine, bio implants, contact lenses and tissue engineering [5–7]. For these and other applications they have the advantage of being biodegradable, as shown in the literature [8–10]. For an extensive review on the preparation and applications of this type of polymers, see [11] and references therein. Kobayashi has described various applications of glycoconjugate polymers in biological and biomedical fields [12].

Sucrose is a carbohydrate feedstock of low molecular weight from which interesting new materials can be elaborated, such as water-soluble and/or biocompatible polymers and other new compounds [1,13–15] owing to its low price. The direct transformation of unprotected sucrose in the context of preparation of derivatives of industrial interest is a challenging task. Our approach was to introduce polymerisable carbohydrate containing moieties into polyvinyl chain polymers [16–19]. In order to avoid the use of protecting groups and to apply more atom-efficient processes, a conventional procedure for the esterification of unprotected sucrose, using triethylamine as a base, was explored. The utilisation of microwave irradiation as a more efficient mode of heating leading to shorter reaction periods was evaluated. The present investigation was extended to the study of the biodegradability of poly(vinylsaccharide)s in culture conditions as a function of time.

## 2. Results and Discussion

### 2.1. Synthesis of Unsaturated Sucrose Esters

**Scheme 1.**
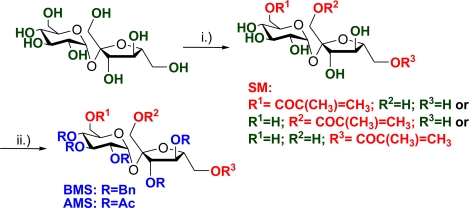
Synthesis of methacryloyl sucrose monomers from unprotected sucrose: **i.)** (CH_3_CH=CHCO)_2_O, py, Et_3_N, 4-DMAP, rt, 44% of **SM** (sucrose methacrylate); **ii.)** BnBr/NaH, DMF, Bu_4_N^+^I^−^, rt, 80% for **BMS** (benzylated methacryl sucrose), and (CH_3_CO)_2_O, py, rt, 93% for **AMS** (acetylated methacryl sucrose).

The procedure adopted for the synthesis of unsaturated sucrose esters was based in the use of the corresponding acyl anhydride in the presence of a base, as a convenient and mild method (Scheme 1). In the case of methacryloyl sucrose ester, the compounds obtained were very reactive and tended to polymerise spontaneously upon concentration. This difficulty was overcome by adding hydroquinone to the mixture, as an inhibitor of free radicals, which was removed prior to the copolymerisation experiments by flash column chromatography [20].

In order to obtain solely monoesters of sucrose, the reaction was monitored by TLC, and stopped at the first appearance of the di-esters. Thus, it was possible to achieve a 44% yield of unsaturated monomethacryloyl esters of sucrose, as a mixture of regioisomers of three monoesters at the primary positions (see ESI- Electronic Supplementary Information).

The esterification reactions were carried out also using H_2_O as solvent, and under microwave irradiation with solvent, which afforded lower yields. Attempts to perform the esterification reaction without solvent under microwave irradiation lead to the octa-methacrylated sucrose. A similar approach to methacryl sucrose (SM) in 20–30% yield has been reported [21].

Hepta-*O*-benzylmonomethacryloyl sucrose (**BMS**) was obtained from the reaction of monomethacrylated sucrose (**SM**) with benzyl bromide in DMF, in the presence of NaH and using Bu_4_N^+^I^−^ as a catalyst. The best yield was obtained when the reagents were mixed at 0 °C, and the mixture was then allowed to warm to room temperature (below 30 °C). After conventional treatment with acetic anhydride in pyridine the corresponding acetylated product (**AMS**) were obtained.

Two useful measures of the potential environmental acceptability of chemical processes are the *E* factor, defined as the mass ratio of waste to desired product, and the atom utilization, calculated by dividing the molecular weight of the desired product by the sum of the molecular weights of all substances produced in the stoichiometric equation [22]. For the mono-esterification of sucrose by direct esterification these values are as follows: *E* factor = 2.32; Atom utilization = 69%. In this calculation the solvent DMF was not taken into consideration as waste as it was easily recovered and reused. According to the study presented by Sheldon in [22], these values are in the range reported for bulk chemicals, and are much lower than for fine chemical industry. These values show that the presented one-step method for valorization of sucrose as renewable natural feedstock is beneficial over the multistep protection-deprotection strategies.

### 2.2. Synthesis of Amphiphilic Copolymers − Copolymerisation of Vinyl Sugar Monomers with Styrene, Methylmethacrylate, α- and β-Pinene by Free Radical Polymerisation

**Scheme 2.**
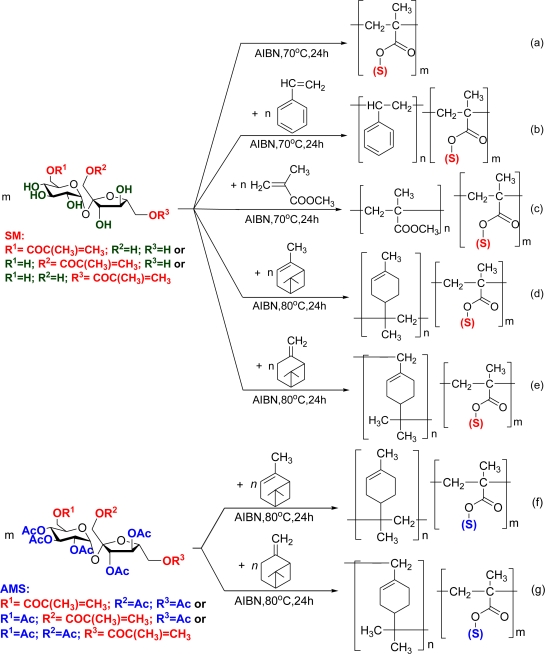

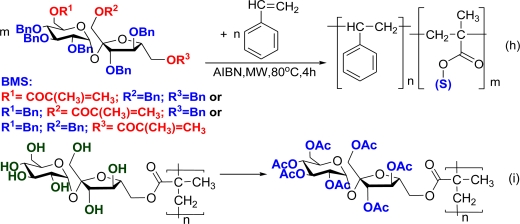
Synthesis of amphiphilic copolymers - copolymerisation of vinyl sugar monomers with styrene, methylmethacrylate, alpha- and beta-pinene. **(a)** Free radical homopolymerisation of sucrose methacrylate (Table 1, entries 5–11). **(b)** Free radical copolymerisation of sucrose methacrylate with styrene (Table 1, entries 1, 2, 12). **(c)** Free radical copolymerisation of sucrose methacrylate with methyl methacrylate (Table 1, entries 3, 4, 13). **(d)** Copolymerisation of sucrose methacrylate with α-pinene (Table 3, entries 19–23; Table 5, entry 29). **(e)** Copolymerisation of sucrose methacrylate with β-pinene (Table 4, entries 24–28; Table 5, entry 30). **(f)** Free radical copolymerisation of α-pinene with hepta-*O-*acetyl-mono-methacryloyl sucrose (Table 6, entries 31–35). **(g)** Free radical copolymerisation of β-pinene with hepta-*O-*acetyl-mono-methacryloyl sucrose (Table 7, entries 36–40). **(h)** Free radical copolymerisation of hepta-*O*-benzylated-mono-*O-*methacryloyl sucrose ester (**BMS**) with styrene (**St**) in the presence of radical initiator AIBN under microwave irradiation (Table 2, entries *14–18*). **(i)** Protection of the OH-groups of the sugar moieties in the polymers, pyridine, 4-DMAP, (CH_3_CO)_2_O, r.t. (Table 8).

Using the monomethacryloyl sucrose esters prepared, several homo- and copolymerization reactions were performed (Scheme 2, Tables 1–7). By increasing the reaction temperature it is possible to achieve a higher incorporation of the sugar in the copolymers, but not higher molecular mass (Table 1). When copolymerization was carried out in aqueous media, higher overall conversion of the monomers was achieved (60–70%). The **SM** monomer was able to polymerize spontaneously at room temperature, giving polymers with high molecular mass, which were hydrophilic and slowly swelled in water. The homopolymer with highest average molecular mass was produced when DMF was used as solvent and at 150 °C (Table 1, entry *6*).

**Figure 1.**
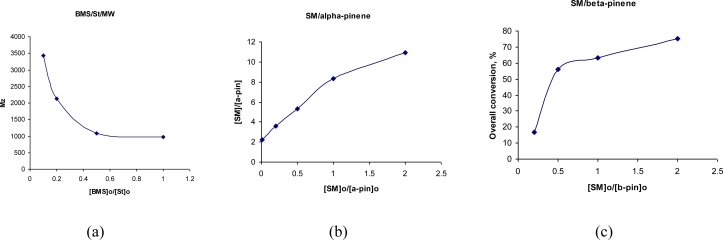
**(a)** Copolymerization of **BMS** with styrene under MW irradiation: dependence of the molecular masses of the copolymers obtained on the initial monomer ratio. **(b)** Copolymerization of **SM** with α-pinene: dependence of the incorporation of sugar in the copolymers obtained on the initial monomer ratio. **(c)** Copolymerization of **SM** with β-pinene: dependence of the overall conversion of the monomers on the initial monomer ratio.

Copolymerization reactions were achieved under microwave irradiation at 80 °C. Under these conditions, the reaction times were reduced from 48 h with conventional heating to only 4 h. The quantity of the solvent used was the minimum needed to homogenize the reaction mixture. Under these conditions a good incorporation of sugar was obtained (3.6 mole ratio), but with a lower molecular mass (1,000-3,000), decreasing when the initial mole sugar ratio increased (Figure 1a).

Copolymers of the hydrophilic sucrose monomer (**SM**) with α- and β-pinene were prepared as a continuation of previous work [17]. α- and β-Pinene are terpene isomers, which are widespread in Nature, mainly in plants as constituents of essential oils [23]. Commercial polymers of these compounds have the common name “terpenic resins” and are obtained by cationic catalysis, generally initiated by Lewis acids [24]. In our case, free radical copolymerisation of unprotected methacryloyl sucrose with pinenes gave water soluble copolymers with low incorporation of pinenes (up to 15 mol%). Based on the sugar content in the copolymer correlated with the initial mole ratio and with the overall conversion of the monomers (Figures 1b,c), we can conclude that the reactivity ratio for sucrose monomer (**SM**) is much higher than that observed for the pinenes, when using radical polymerisation. On the other hand, the protected methacryloyl sucrose monomers and pinenes showed comparable reactivity ratios when using a free radical initiator and copolymers with reasonable incorporation of sugar were obtained (Tables 6 and 7).

It is known that cationic polymerisation of pinenes is a fast process leading to polymers with higher molecular mass then the corresponding radical polymerisation [25]. Our attempt to copolymerise pinenes with BMS-monomer by a cationic mechanism resulted in polymer consisting mainly of polypinenes, what can be explained by the much lower reactivity ratio of the protected sugar. Therefore we opted to experiment the cationic copolymerisation between unprotected methacryloyl sucrose and pinenes, expecting they would show comparable reactivity ratios. This prevision was confirmed (Table 5), giving copolymers with a sugar content similar to the initial monomer ratio.

Some of the polymers obtained were not soluble in organic solvents (dichloromethane, chloroform, toluene, acetone) and very poorly soluble in water. This made their characterization by NMR spectroscopy and SEC difficult. In order to overcome the problems of obtaining soluble samples for analysis, we have carried out experiments involving protection of the sugar moieties in the polymers by polymer-analogous reactions [Scheme 2(i) and Table 8], and the resulting materials were characterized by NMR, SEC, DSC, AFM, XPS and TOF-SIMS (see ESI). In Table 8 data for four acetylated polymer samples is presented. The acetylated polymer **42** was used for comparison, as it was originated from the soluble polymer **1**, with relatively low content of SM. In this case, after acetylation we observed an increase of the average stoichiometric molecular mass corresponding to the expected introduction of the acetyl groups. Polymers **3** and **7** upon acetylation showed significantly higher values then stoichiometric increase of the molecular mass, which are probably due to the dissolution of the unsoluble fraction; in the case of the polymer **22**, containing pinene units, partial degradation during the acetylation procedure must have occurred.

### 2.3. Biodegradation Studies: Incubation in Culture

The visual growth rating [26] test is valuable in assessing the performance of a polymer during its use under such conditions. The samples were used as the sole carbon source for the fungus, e.g., *A. niger*. During incubation with fungal culture, the spores (black spots) as colonies were observed to grow. Tables 2–6 ESI represent the data of fungal colonization/fungal growth (visual growth) on the surface of polymer film after 90 days of culture incubation but the initiation of fungal growth could be seen by microscope within 15–20 days. The absence of any colonization in a controlled Petri dish (without polymer sample) clearly suggested that fungus is using the polymer specimen as a sole source of carbon, as there was complete absence of carbon in nutrient agar. It could be observed that with increasing time of incubation, the fungal colonization was found to increase. The higher colonization could be attributed to easy consumption of short chains as energy source by fungus with increasing incubation time in culture which concludes that the copolymer was bio-assimilated during microbial attack.

**Figure 2.**
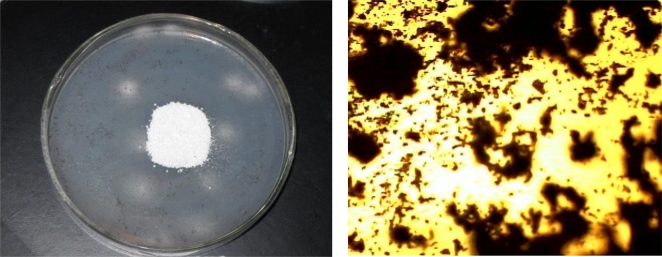
Photograph after 90 days of incubation of sample in fungal culture. The fungal growth (≥25%) indicates the biodegradability of the sample *41/*Table 8: poly(acetyl methacryloyl sucrose-co-styrene).

The photographs (Figures 2 and 12-14-ESI) of the cultured sample showed the eroded surface with some residue. The microbes’ adhesion onto the surface was quite evident after 30 days of incubation. Once microbes have been adsorbed, their penetration into the polymer was rapid as they consumed it.

The degraded polymer structure was examined by means of Polar Optical Microscopy (Olympus B201). All optical micrographs presented were obtained under cross-polarized light. The tested polymer samples have been eaten by microorganisms with respect to their biodegradability, *i.e.*, the dark region is the mixture of microorganisms with polymer sample, the degradable part (Figure 2 and 12-14-ESI).

Four different copolymer samples were tested by this procedure: **2**/Table 1: poly(methacryloyl sucrose-co-styrene), bearing unprotected sucrose moieties; **41**/Table 8: poly(acetyl methacryloyl sucrose-co-styrene), bearing protected by acetyl groups sucrose moieties; and copolymers bearing protected by benzyl groups sucrose moieties, synthesized as described in [17]: *poly*(*1′*,*2*,*3*,*3′*,*4*,*4′*,*6-hepta-O-benzyl-6′-O-methacryloyl-sucrose*-co*-*α*-pinene*) and *poly*(*1′*,*2*,*3*,*3′*,*4*,*4′*,*6-hepta-O-benzyl-6′-O-methacryloyl-sucrose*-co-*β-pinene*). As expected, the copolymers with unprotected sucrose moieties showed higher biodegradability, but as a very interesting result, the copolymers with protected sucrose moieties also showed the capability to biodegrade, although slower. This could be as a result of hydrolization reactions taking place during the incubation period, and is very promising for future applications of this type of biodegradable polymers (Figure 2).

After fungal degradation of the sugar fraction the remaining lower molecular weight polystyrene fragments were tested for toxicity. No toxic effects were observed on earthworms living in soil treated with the oligo-styrene fragments. Similarly, fish were not affected by the presence of the fragments in the tank for an extended period. The oligo-styrene fragments mixed in soil were also put in cage containing rats; also no toxic effects were observed.

## 3. Experimental Section

### 3.1. General Methods

Reagents and solvents were purified before use [27]. NMR spectra were recorded at 400 MHz on a Bruker AMX-400 instrument in CDCl_3_ with chemical shift values (δ) reported in ppm downfield from TMS. Size Exclusion Chromatography (SEC) results were obtained using light scattering, refractive index, ultra-violet and viscometer detectors in a temperature controlled oven (set at 35 °C) coupled to a integrated solvent and sample delivery module (degasser, pump and auto sampler), at a flow rate 1 mL/min, using DMF as eluent, and with an injection volume of 150 μL. The detectors alignment and instrument sensitivity parameters were previously calibrated using a narrow molecular weight polystyrene standard.

X-Ray Photoelectron Spectroscopy (XPS): Analysis of the samples were performed using an VG Escalab 250 iXL ESCA instrument (VG Scientific), equipped with aluminum Ka1,2 monochromatized radiation at 1486.92 eV X-ray source. Due the non conductor nature of samples it was necessary to use an electron flood gun to minimize surface charging. Neutralization of the surface charge was performed by using both a low energy flood gun (electrons in the range 0 to 14 eV) and an electrically grounded stain steel screen placed directly on the sample surface. The XPS measurements were carried out using monochromatic Al-Kα radiation (*hν* = 1486.92 eV). Photoelectrons were collected from a takeoff angle of 90°relative to the sample surface. The measurement was done in a Constant Analyser Energy mode (CAE) with a 100 eV pass energy for survey spectra and 20 eV pass energy for high resolution spectra.

Charge referencing was done by setting the lower binding energy C 1s photopeak at 285.0 eV C1s hydrocarbon peak (1). The spectra fitting is based on “Chi-squared” algorithm used to determine the goodness of a peak fit. Chi-squared < 2 implies a good fit. The components of the peaks can be free or coupled of ways reflecting the chemistry of the sample. In most of the cases the FWHM value was fixed to defined values. Analysis Conditions: X-Ray Source: Al Ka Monochromatic 1486.92 eV; Take off Angle: 90° (relative sample surface); Mode: CAE; Spot size: 500 um; Flood Gun: Active; Pressure: 4 × 10 -10 mbar; Survey Spectra: 100 eV pass Energy; High Resolution spectra: 20 eV pass Energy.

The mass spectra of the samples were recorded on a TOF-SIMS IV instrument from Ion-Tof GmbH (Germany). The sample was bombarded with a pulsed gallium ion beam. The secondary ions generated were extracted with a 10 KV voltage and their time of flight from the sample to the detector, was measured in a reflectron mass spectrometer. Typical analysis conditions for this work were: Analysis parameters Ion Beam: 69Ga+; PI dose: 3 × 1011 ions/cm^2^; Mode: Low Current; Energy: 25 KeV; Current: 0.5 pA; Area: 500 × 500 um^2^; Flood Gun : ACTIVE.

### 3.2. Synthesis of Monomers

*Sucrose Methacrylate* (**SM**): To a 0.1 M solution of sucrose (0.500 g, 1.46 mmol) in anhyd. DMF, Et_3_N (0.148 g, 1.46 mmol) and a catalytic amount of 4-DMAP was added. The mixture was cooled to 0 °C, and then a solution of methacrylic anhydride (0.225 g, 1.46 mmol) in DMF (1 mL) was added. The reaction mixture was allowed to warm to r.t. The reaction was followed by TLC, and at first appearance of the diester as a faster moving fraction, it was stopped by evaporation of the solvent. Purification by flash column chromatography, eluent ethyl acetate-acetone-water, 10:10:1, yielded 0.263 g (44%) of **SM**. ^1^H-NMR (D_2_O): δ = 6.15-6.05(1H, =C*H*α), 5.75-5.63(1H, =C*H*β), 5.47 (d, *J*_1,2_ = 3.4Hz, H-1), 5.36 (d, *J*_1,2_ = 3.7Hz), 5.29 (d, *J*_1,2_ = 3.3Hz, H-1), 4.33 (m, 1H, H-3′), 4.11 (m, 1H, H-5), 4.01 (m, 1H, H-4′), 3.92 (m, 1H, H-5′), 3.71 (m, 5H, H-6, H-6′, H-3), 3.58 (m, 2H, H-1′), 3.42 (m, 2H, H-2, H-4), 1.84(s, 3H, C*H*_3_); ^13^C-NMR (D_2_O): δ = 169.7, 169.1, 169.0 (*C*OO), 135.9, 135.7 (quat *C*=), 128.3, 127.9, 127.6 (=*C*H_2_), 104.4, 104.1, 102.8 (*C*-2′), 93.1, 92.6, 92.4, 89.9 (*C*-1), 82.7, 81.9, 81.8, 78.8, 78.3, 77.2, 76.8, 76.7, 75.8, 75.1, 74.5, 74.4, 74.2, 73.8, 73.3, 73.2, 72.9, 72.7, 71.5, 71.3, 70.8, 70.7, 70.0, 69.7, 69.6, 69.5 (7*C*H-sucrose skeleton), 66.4, 64.0, 63.3, 63.0, 62.8, 62.3, 61.8, 61.6, 61.3, 60.7, 60.5, 60.3 (3*C*H_2_-sucrose skeleton), 17.7 (*C*H_3_). This assignment has been confirmed by DEPT, COSY, and HMQC.

*Acetylated Methacryl Sucrose* (**AMS**): To a solution of **SM** (0.150 g, 0.365 mmol) in anhyd. pyridine (10 mL) was added acetic anhydride (0.520 g, 5.11 mmol) slowly at 0 °C. The reaction mixture was allowed to warm to r.t. and was stirred overnight. The solvent was dist. off at reduced pressure, and the residue was purified at flash column chromatography, eluent: hexane-ether, 3:1. ^1^H-NMR (CDCl_3_): δ = 6.17 (d, *J =* 13.6 Hz, 1H, =C*H*α), 5.70 (d, *J*_1,2_ *=* 3.6 Hz, 1H, H-1), 5.62 (d, *J =* 10.8 Hz, 1H, =C*H*β), 5.44 (t+d, *J*_2-3-4_ = 9.9 Hz, *J*_3′-4′_ = 4.8 Hz, 2H, H_3_, H_3′_), 5.38 (t, *J*_3′-4′-5′_ = 4.8 Hz, 1H, H_4′_), 5.13 (t, *J*_3-4-5_ = 9.8 Hz, 1H, H_4_), 4.82 (dd, *J*_1-2_ = 3.7 Hz, *J*_2-3_ = 10.4 Hz, 1H, H_2_), 4.43-4.17 (m, 8H, H_5_, H_6′_, H_6_, H_5′_, H_1′_), 2.18 (s, 3H, C*H*_3_), 2.11 (s, 3H, C*H*_3_), 2.11 (s, 3H, C*H*_3_), 2.09 (s, 3H, C*H*_3_), 2.03 (s, 3H, C*H*_3_), 2.02 (s, 3H, C*H*_3_), 1.97 (s, 3H, C*H*_3_), 1.96 (s, 3H, C*H*_3_); ^13^C-NMR (CDCl_3_): 170.5-169.4 (8-COO−), 135.9 (COO(CH_3_)*C*=), 127.6 (*C*H_2_=), 104.1 (C_2′_), 90.0 (C_1_), 79.2 (C_5′_), 75.8 (C_3′_), 75.1 (C_4′_), 70.3 (C_2_), 69.8 (C_3_), 68.6 (C_5_), 68.4 (C_4_), 63.6 (C_6′_), 62.9 (C_1′_), 60.7 (C_6_), 20.6 (7*C*H_3_CO), 17.8 (CH_3_).

*Benzylated methacryl sucrose* (**BMS**): **SM** (0.150 g, 0.365 mmol) was dissolved in DMF (10 mL) together with a catalytic amount (5 mg) of (n-Bu)_4_N^+^I^−^. The solution was cooled to 0 °C, and NaH (50% suspension in oil, 0.196 g, 4.10 mmol) was added carefully. After 20 min benzyl bromide (0.701 g, 4.10 mmol) was added dropwise for 15 min. The ice bath was removed and the reaction monitored by TLC (hexane-ethyl acetate, 5:1). When there was no more of the initial compound (4–5 hours), the reaction mixture was poured into cold H_2_O (50 mL). The product was extracted four times with diethyl ether (20 mL), and the combined organic layers washed twice with H_2_O (10 mL), dried with Na_2_SO_4_ and concentrated. The residue was purified by column chromatography, eluent hexane-ethyl acetate, 5:1, to yield 0.304 g (80%) of **BMS**. ^1^H-NMR (CDCl_3_): δ = 7.30 (m, 35H, Ar-H), 6.10 (s, 1H, =C*H*α), 5.78 (d, 1H, *J*_1,2_ = 3.4 Hz, H-1), 5.54 (s, 1H, =C*H*β), 4.87 (t, 2H, *J* = 11.4 Hz, Ar-C*H*_2_), 4.54 (m, 13H, H-3′, H-4′, H-6, H-6′, H-1′, Ar-C*H*_2_), 4.27 (t, 1H, *J*_3,4,5_ = 7.6 Hz, H-4), 4.17 (t, 2H, *J* = 12.6Hz, Ar-C*H*_2_), 4.04 (m, 2H, H-5, Ar-C*H*_2_), 3.93 (t, 1H, *J*_2,3,4_ = 9.2Hz, H-3), 3.70 (d, 2H, *J* = 10.9Hz, Ar-C*H*_2_), 3.64 (m, 1H, H-5′), 3.52 (d, 2H, *J* = 9.7Hz, Ar-C*H*_2_), 3.39 (dd, 1H, *J*_1,2_ = 3.5Hz, *J*_2,3_ = 9.5Hz, H-2), 1.92 (s, 3H, C*H*_3_); ^13^C-NMR (CDCl_3_): 169.9 (*C*OO−),138.6, 138.2, 138.1, 137.9, 137.7 (C_q_ benzyl groups), 135.5 (COO(CH_3_)*C*=),128.4, 127.9, 127.8, 127.6 (C_Ar_), 127.2 (*C*H_2_=), 105.2 (C-2′), 90.3 (C-1), 84.0, 83.3, 81.4, 80.5, 79.7, 78.1, and 70.1 (C-2,3,3′,4,4′,5,5′), 75.5, 75.1, 73.5, 73.1, 72.5, 72.4, 70.7 (C-1′ + 6 OCH_2_Ph), 63.6, 62.9 (C-6,6′), 17.8 (CH_3_).

### 3.3. General Procedure for Copolymerisation under Mild Conditions

Copolymerisations of sucrose methacrylate (SM) with styrene or methyl methacrylate were carried out in anhyd. DMF solutions (0.1 M) in the presence of AIBN as radical initiator (1% by weight with respect to the monomer mixture). Dissolved oxygen was removed from the solutions by three freeze-thaw cycles on the vacuum pump. They were then heated at 70 °C until the polymerisations were complete and the solutions were then cooled to r.t. and the product precipitated in cold acetone. The white solids were filtered and washed several times with cold acetone. The polymers were purified by repeated dissolution in toluene and reprecipitation in cold acetone and dried under vacuum.

### 3.4. General Procedure for Copolymerisation in an Autoclave

The autoclave copolymerisations of **SM** with styrene or methyl methacrylate were carried out in anhyd. DMF solution (0.1 M) in the presence of AIBN as radical initiator (1% by weight with respect to the monomer mixture). The solutions were placed in an autoclave vessel and kept at 150 °C for 24 h. The solutions were then cooled to r.t. and 0.05 mL samples were taken from the mixture and dropped into acetone. If there was no precipitation, the mixture was heated for another 24 h. As soon as precipitation appeared, the reaction was stopped by precipitating the whole mixture in cold acetone. The white solids were filtered and washed several times with cold acetone. The polymers were purified by repeated dissolution in toluene and reprecipitation in cold acetone and finally dried under vacuum.

### 3.5. General Procedure for Copolymerisation in Aqueous Media

To a solution of *O*-methacryloyl sucrose (1.0 g, 2.44 mmol) in H_2_O were added styrene or methyl methacrylate (8.13 mmol, 0.847 g of styrene or 0.814 g of methyl methacrylate) and a phase transfer catalyst Bu4N^+^I^−^. AIBN (0.02 g) was dissolved in acetone (1 mL) and added to the reaction mixture. The solutions were flushed with Ar, and stirred at 60 °C. After cooling, the solutions were concentrated, and precipitated in cold EtOH. The white solids were filtered, washed, and dried.

### 3.6. General Procedure for Polymerisation and Copolymerisation in Focused Microwave Oven

The reactions were performed in MicroSynth Labstation (MileStone, USA) in closed vials. To the monomer mixture with a minimum amount of solvent (dry toluene) the initiator of polymerisation AIBN was added under positive pressure of dry Ar. The vials were closed and the temperature probe was inserted in a reference vial. The desired temperature/potency/ time protocol was programmed – to work at constant temperature of 80 °C (when the program was varying the power). When the polymerisations were complete, the samples were cooled to r.t. and the products precipitated in cold EtOH. The white solids were filtered and washed several times with cold EtOH. The powder polymers were purified by repeated dissolution in toluene and reprecipitation in cold EtOH and dried under vacuum.

### 3.7. General Procedure for Cationic Homopolymerisation of α- and β-Pinene

Solutions of the monomer SM and α- or β-pinene in dry DMF (0.1 M) were cooled to 0 °C, and AlCl_3_ (5 weight%) were added under positive pressure of dry Ar. Stirred for additional 24 hours at r.t., and the reaction mixtures were precipitated in cold MeOH. The white solids were filtered and washed several times with cold MeOH. The powder polymers were purified by repeated dissolution in DMF and reprecipitation in cold MeOH and dried under vacuum.

### 3.8. General Procedure for Radical Copolymerisation in Solution

Copolymerisations of the sucrose derivatives SM and AMS with α- and β-pinene were carried out in pyridine solutions (0.1 M) in the presence of AIBN as radical initiator (1% by weight with respect to the monomer mixture). The solutions were heated at 80 °C until the polymerisations were complete, and the solutions were then cooled to r.t. and the product precipitated in cold MeOH. The white solids were filtered and washed several times with cold MeOH. The powder polymers were purified by repeated dissolution in toluene and reprecipitation in cold MeOH and dried under vacuum.

### 3.9. General Procedure for Polymer-Analogous Reactions

The corresponding polymer samples were dissolved in pyridine, and the calculated amount of acetic anhydride in two fold excess was added under Ar. The solutions were stirred for additional at r.t., and precipitated in cold MeOH. The white solids were filtered and washed several times with cold MeOH. The powder polymers were purified by repeated dissolution in DMF and reprecipitation in cold MeOH, and dried under vacuum.

### 3.10. Culture Incubation

The test fungi (*Aspergillus niger*) was obtained from the Biochemistry Division, National Chemical Laboratory, Pune (India), and nutrient salt agar was prepared by dissolving potassium dihydrogen phosphate (0.700 g), magnesium sulfate (0.700 g), ammonium nitrate (1.0 g), sodium chloride (0.005 g), ferrous sulfate (0.002 g), manganese sulfate (0.001 g), and agar (15.00 g) in 1 L of distilled water. After the medium was sterilized at 120 ± 5 °C for 25 min, the pH was adjusted between 6.5 and 7.0 by the addition of a 0.1 N solution of NaOH. For providing the solidified agar layer (depth 4–7 mm) nutrient salt was poured into sterilized Petri dish. Then, the test specimen, (as powder) was spread (3 × 3 cm) on the surface of the agar layer and inoculated by spraying the spore suspension. The petri-dishes were incubated at 28–30 °C after being sealed by wax to avoid any further contamination. The growth fungal colonies were quantified periodically. Morphological changes of the culture incubated sample were also studied by optical microscope (Olympus, Model B X 50 F4, Japan).

## 4. Conclusions

In summary, three unsaturated esters of sucrose were synthesised directly from unprotected sucrose, in good yields, using a simple and mild known procedure. These methacryloyl sugar derivatives have been copolymerised with styrene, methyl metacrylate, or α- and β-pinenes, by a free radical or cationic process, in organic or aqueous media, by conventional heating or under microwave irradiation, to yield unbranched linear polymer materials with pendant sucrose moieties. The characterisation of the polymer products by NMR, SEC, AFM, XPS, TOF-SIMS, enabled an assessment of structural, thermal, and rheological information. The higher incorporation of the sucrose monomer was achieved using methacryloyl sucrose monomer. The use of abundant renewable non-petroleum based starting materials for the production of novel polymeric materials has been demonstrated. The optimisation of the polymerisation procedure with a view to possible applications, as well as the biodegradability of copolymers, containing sucrose moieties side groups, was also investigated. Biodegradation tests of the polymer samples bearing unprotected sucrose moieties by a microbial fungal culture (*Aspergillus niger*) method showed fungal growth ≥ 60%, which indicated the good biodegradability of the samples, with bio-assimilation during microbial attack. Another interesting result, opening possibilities for future applications, was that the copolymers with protected sucrose moieties showed to be biodegrade, although slower that the corresponding with not protected sucrose moieties.

## Electronic Supplementary Information (ESI) Available:

Proton and carbon NMR spectra of reported compounds; and results from: Size Exclusion Chromatography (SEC); Differential Scanning Calorimetry (DSC); Atomic Force Microscopy (AFM); X-ray photoelectron spectroscopy (XPS); Time of Flight – Secondary Ion Mass Spectroscopy (TOF-SIMS); Biodegradation studies - incubation in culture.

## Figures and Tables

**Table 1.** Free radical homo- and copolymerisation of sucrose methacrylate (**SM**) with styrene (**St**) or methyl methacrylate (**MM**).

**№**	**Sugar**	**Alkene**	$\frac{{\left[\text{sug}\right]}_{0}}{{\left[\text{alk}\right]}_{0}}$^*a*^	$\frac{\left[\text{sug}\right]}{\left[\text{alk}\right]}$^*b*^	**Solvent, initiator**	**Reaction temp.**	**Reaction time [h]**	**Yield^c^ [%]**	**M**_**n**_**^d^[g/mol]**	**M**_**W**_**^d^[g/mol]**	**M**_**n**_**/M**_**W**_**^d^**	**φ ^e^ [Å]**	**D^f^[cm2/s]**
1	SM	St	1	0.06	DMF, AIBN	70 °C	48	26.5	33970	40760	1.2	745	4.90 × 10^−9^
2	SM	St	1	0.34	DMF, AIBN	150°C	48	25.0	7800	12470	1.6	401	9.09 × 10^−9^
3^g^	SM	MM	1	0.96	DMF, AIBN	70 °C	48	32.0	69180	131450	1.9	1370	2.66 × 10^−9^
4	SM	MM	1	0.35	DMF, AIBN	150 °C	48	23.2	19200	29795	1.5	631	5.77 × 10^−9^
5	SM	_	100%	100%	DMF, AIBN	70 °C	48	59.4	246800	468920	1.9	2648	1.37 × 10^−9^
6	SM	_	100%	100%	DMF, AIBN	150 °C	48	44.7		insoluble			
7^g^	SM	_	100%	100%	_	r.t.	24	100	29370	38180	1.3	718	5.07 × 10^−9^
8	SM	_	100%	100%	DMF	r.t.	48	12.8	53400	85460	1.6	1092	3.33 × 10^−9^
9	SM	_	100%	100%	H_2_O	r.t.	48	13.9		insoluble			
10	SM	_	100%	100%	pyridine	r.t.	24	23.7	20400	28550	1.4	617	5.90 × 10^−9^
11	SM	_	100%	100%	H_2_O, AIBN	60 °C	48	27.8	48030	81650	1.7	1066	3.41 × 10^−9^
12	SM	St	0.3	0.07	H_2_O+ acetone, AIBN	60 °C	48	71.4	17770	23095	1.3	553	6.69 × 10^−9^
13	SM	MM	0.3	0.12	H_2_O+ acetone, AIBN	60 °C	48	59.0	12150	17430	1.4	_	_

**Table 2.** Free radical copolymerisation of hepta-*O*-benzylated-mono-O-methacryloyl sucrose ester (**BMS**) with styrene (**St**) in the presence of radical initiator AIBN under MW irradiation (toluene, 80 °C).

**№**	**Sugar**	**Alkene**	$\frac{{\left[\text{sug}\right]}_{0}}{{\left[\text{alk}\right]}_{0}}$^*a*^	$\frac{\left[\text{sug}\right]}{\left[\text{alk}\right]}$^*b*^	**Solvent, initiator**	**Reaction temp.**	**Reaction time [h]**	**Yield^c^[%]**	**M**_**n**_**^d^[g/mol]**	**M**_**W**_**^d^[g/mol]**	**M**_**n**_**/M**_**W**_**^d^**	**φ ^e^ [Å]**	**D^f^[cm2/s]**
14a	_	St	0	0	toluene, AIBN	MW, 80 °C	4	87	5300	9370	1.8	_	_
14b	BMS	_	100%	100%	toluene, AIBN	MW, 80 °C	4	0.7	33400	56820	1.7	883	4.12 × 10^−9^
15	BMS	St	1	0.29	toluene, AIBN	MW, 80 °C	4	55.7	880	970	1.1	106	3.44 × 10^−8^
16	BMS	St	0.5	3.57	toluene, AIBN	MW, 80 °C	4	7.4	980	1080	1.1	112	3.25 × 10^−8^
17	BMS	St	0.2	0.31	toluene, AIBN	MW, 80 °C	4	34.9	1760	2120	1.2	159	2.29 × 10^−8^
18	BMS	St	0.1	0.11	toluene, AIBN	MW, 80 °C	4	62.8	3130	3440	1.1	205	1.78 × 10^−8^

**Table 3.** Free radical copolymerisation of sucrose methacrylate (**SM**) with α-pinene (α-P).

**№**	**Sugar**	**Alkene**	$\frac{{\left[\text{sug}\right]}_{0}}{{\left[\text{alk}\right]}_{0}}$^*a*^	$\frac{\left[\text{sug}\right]}{\left[\text{alk}\right]}$^*b*^	**Solvent, initiator**	**Reaction temp.**	**Reaction time [h]**	**Yield^c^[%]**	**M**_**n**_**^d^[g/mol]**	**M**_**W**_**^d^[g/mol]**	**M**_**n**_**/M**_**W**_**^d^**	**φ ^e^ [Å]**	**D^f^[cm2/s]**
19	SM	α-P	2	10.9	py, AIBN	80 °C	48	13.7	12250	19600	1.6	_	_
20	SM	α-P	1	8.31	py, AIBN	80 °C	48	72.6	26600	45300	1.7	_	_
21	SM	α-P	0.5	5.29	py, AIBN	80 °C	48	60.7	21330	33000	1.6	_	_
22	SM	α-P	0.2	3.57	py, AIBN	80 °C	48	39.8	15600	23400	1.5	_	_
23	SM	α-P	0.01	2.22	py, AIBN	80 °C	48	25.0	12450	18700	1.5	_	_

**Table 4.** Free radical copolymerisation of sucrose methacrylate (**SM**) with β-pinene (β-P).

**№**	**Sugar**	**Alkene**	$\frac{{\left[\text{sug}\right]}_{0}}{{\left[\text{alk}\right]}_{0}}$^*a*^	$\frac{\left[\text{sug}\right]}{\left[\text{alk}\right]}$^*b*^	**Solvent, initiator**	**Reaction temp.**	**Reaction time [h]**	**Yield^c^[%]**	**M**_**n**_**^d^[g/mol]**	**M**_**W**_**^d^[g/mol]**	**M**_**n**_**/M**_**W**_**^d^**	**φ ^e^ [Å]**	**D^f^[cm2/s]**
24	SM	β-P	2	7.54	py, AIBN	80 °C	48	75.0	5980	10760	1.8	_	_
25	SM	β-P	1	7.32	py, AIBN	80 °C	48	63.1	8110	12980	1.6	_	_
26	SM	β-P	0.5	8.34	py, AIBN	80 °C	48	56.1	5140	9760	1.9	_	_
27	SM	β-P	0.2	3.06	py, AIBN	80 °C	48	17.0	10800	17230	1.6	_	_
28	SM	β-P	0.01	6.13	py, AIBN	80 °C	48	18.3	11650	19800	1.7	_	_

**Table 5.** Cationic copolymerisaton of sucrose methacrylate (**SM**) with α- and β-pinenes.

**№**	**Sugar**	**Alkene**	$\frac{{\left[\text{sug}\right]}_{0}}{{\left[\text{alk}\right]}_{0}}$^*a*^	$\frac{\left[\text{sug}\right]}{\left[\text{alk}\right]}$^*b*^	***Solvent*,*initiator***	**Reaction temp.**	**Reaction time [h]**	**Yield^c^[%]**	**M**_**n**_**^d^[g/mol]**	**M**_**W**_**^d^[g/mol]**	**M**_**n**_**/M**_**W**_**^d^**	**φ ^e^ [Å]**	**D^f^[cm2/s]**
29	SM	α-P	0.1	0.14	*DMF*, *AlCl_3_*	r.t.	24	35.0	4400	7504	1.7	_	_
30	SM	β-P	0.1	0.12	*DMF*, *AlCl_3_*	r.t.	24	29.1	3700	5560	1.5	_	_

**Table 6.** Free radical copolymerisation of hepta-O-acetylated-mono-O-methacryloyl sucrose ester (**AMS**) with α-pinene (**α-P**).

**№**	**Sugar**	**Alkene**	$\frac{{\left[\text{sug}\right]}_{0}}{{\left[\text{alk}\right]}_{0}}$^*a*^	$\frac{\left[\text{sug}\right]}{\left[\text{alk}\right]}$^*b*^	**Solvent, initiator**	**Reaction temp.**	**Reaction time [h]**	**Yield^c^[%]**	**M**_**n**_**^d^[g/mol]**	**M**_**W**_**^d^[g/mol]**	**M**_**n**_**/M**_**W**_**^d^**	**φ ^e^ [Å]**	**D^f^[cm2/s]**
31	AMS	α-P	2	1.53	pyridine, AIBN	80 °C	48	9.37	1970	2170	1.1	_	_
32	AMS	α-P	1	0.63	pyridine, AIBN	80 °C	48	19.8	2150	2370	1.1	_	_
33	AMS	α-P	0.5	0.35	pyridine, AIBN	80 °C	48	8.02	2290	2750	1.2	_	_
34	AMS	α-P	0.2	0.12	pyridine, AIBN	80 °C	48	5.72	2380	2620	1.1	_	_
35	AMS	α-P	0.1	0.07	pyridine, AIBN	80 °C	48	13.2	2080	2500	1.2	_	_

**Table 7.** Free radical copolymerisation of hepta-*O*-acetylated-mono-*O*-methacryloyl sucrose ester (**AMS**) with β-pinene (**β-P**).

**№**	**Sugar**	**Alkene**	$\frac{{\left[\text{sug}\right]}_{0}}{{\left[\text{alk}\right]}_{0}}$^*a*^	$\frac{\left[\text{sug}\right]}{\left[\text{alk}\right]}$^*b*^	**Solvent, initiator**	**Reaction temp.**	**Reaction time [h]**	**Yield^c^[%]**	**M**_**n**_**^d^[g/mol]**	**M**_**W**_**^d^[g/mol]**	**M**_**n**_**/M**_**W**_**^d^**	**φ ^e^ [Å]**	**D^f^[cm2/s]**
36	AMS	β-P	2	1.21	pyridine, AIBN	80 °C	48	21.4	1700	1880	1.1	_	_
37	AMS	β-P	1	0.34	pyridine, AIBN	80 °C	48	13.4	1850	2400	1.3	_	_
38	AMS	β-P	0.5	0.22	pyridine, AIBN	80 °C	48	24.6	2200	2640	1.2	_	_
39	AMS	β-P	0.2	0.95	pyridine, AIBN	80 °C	48	8.38	2150	2370	1.1	_	_
40	AMS	β-P	0.1	0.02	pyridine, AIBN	80 °C	48	3.89	1970	2170	1.1	_	_

[a] Initial (feed) mol ratio of the monomers.

[b] Mole ratio of the comonomers in the copolymer, determined by ^1^H-NMR.

[c] Overall conversion of the monomers.

[d] Measured by SEC.

[e] Effective diameter, determined by light-scattering.

[f] Diffusion coefficient, determined by light-scattering.

[g] The polymers are partially soluble and the data presented express only the behaviour of the soluble fraction.

**Table 8.** Polymer analogous acetylation of hydroxyl groups in the sugar moieties with acetic anhydride (CH_3_CO)_2_O in pyridine, at r.t.

**№**	**Copolymer**	**Reaction time [h]**	**Yield^a^(%)**	**M**_**n**_**^b^[g/mol]**	**M**_**W**_**^b^[g/mol]**	**M**_**n**_**/M**_**W**_**^b^**	φ ^c^ [Å]	**D^d^[cm^2^/s]**
41	Table 1, pol. 7	48	40.1	98700	148040	1.5	1454	2.50 × 10^−9^
42	Table 1, pol. 1	120	67.3	38200	49660	1.3	824	4.42 × 10^−9^
43	Table 1, pol. 3	120	67.1	123000	233740	1.9	1852	1.97 × 10^−9^
44	Table 3, pol. 22	120	81.8	11800	18830	1.6	497	7.33 × 10^−9^

[a] Overall conversion of the monomers.

[b] Measured by SEC.

[c] Effective diameter, determined by light-scattering.

[d] Diffusion coefficient, determined by light-scattering.
